# The Contribution of TRPM8 and TRPA1 Channels to Cold Allodynia and Neuropathic Pain

**DOI:** 10.1371/journal.pone.0007383

**Published:** 2009-10-08

**Authors:** Ombretta Caspani, Sandra Zurborg, Dominika Labuz, Paul A. Heppenstall

**Affiliations:** Klinik für Anaesthesiologie und operative Intensivmedizin, Charité Universitätsmedizin Berlin, Campus Benjamin Franklin, Berlin, Germany; Center for Genomic Regulation, Spain

## Abstract

Cold allodynia is a common feature of neuropathic pain however the underlying mechanisms of this enhanced sensitivity to cold are not known. Recently the transient receptor potential (TRP) channels TRPM8 and TRPA1 have been identified and proposed to be molecular sensors for cold. Here we have investigated the expression of TRPM8 and TRPA1 mRNA in the dorsal root ganglia (DRG) and examined the cold sensitivity of peripheral sensory neurons in the chronic construction injury (CCI) model of neuropathic pain in mice.

In behavioral experiments, chronic constriction injury (CCI) of the sciatic nerve induced a hypersensitivity to both cold and the TRPM8 agonist menthol that developed 2 days post injury and remained stable for at least 2 weeks. Using quantitative RT-PCR and in situ hybridization we examined the expression of TRPM8 and TRPA1 in DRG. Both channels displayed significantly reduced expression levels after injury with no change in their distribution pattern in identified neuronal subpopulations. Furthermore, in calcium imaging experiments, we detected no alterations in the number of cold or menthol responsive neurons in the DRG, or in the functional properties of cold transduction following injury. Intriguingly however, responses to the TRPA1 agonist mustard oil were strongly reduced.

Our results indicate that injured sensory neurons do not develop abnormal cold sensitivity after chronic constriction injury and that alterations in the expression of TRPM8 and TRPA1 are unlikely to contribute directly to the pathogenesis of cold allodynia in this neuropathic pain model.

## Introduction

Neuropathic pain is a debilitating condition that is poorly understood and often untreatable. It is initiated by damage to the nervous system and is characterized by the emergence of spontaneous pain (i.e. pain that occurs in the absence of stimulation), hyperalgesia (an increased sensitivity to noxious stimuli), and allodynia (pain evoked by normally innocuous stimuli) [1]. A common complaint of neuropathic pain patients is an increased sensitivity to cold temperatures, or cold allodynia [2]–[4]. This symptom, which is also observed in animal models of neuropathic pain [5], leads to pain and discomfort at temperatures that are normally perceived as being innocuously cool. Despite the importance and prevalence of cold allodynia, little is known about the underlying molecular mechanisms.

Recently, much progress has been made in our understanding of cutaneous thermosensation and of the thermal transduction mechanisms intrinsic to peripheral sensory neurons [6]. Several candidate thermosensor molecules have been identified belonging to the Transient Receptor Potential (TRP) ion channel family [7]. Two of these ion channels, termed TRPM8 and TRPA1, have been proposed to function as cold transducers [8], [9]. TRPM8 is expressed by a small population of cold-sensitive sensory neurons and is activated at cool temperatures (∼26°C) and by the cooling compound menthol [10], [11]. Mouse knockout studies have revealed that TRPM8 is required for cold sensation over a broad range of innocuous and noxious cold temperatures [12]–[14]. TRPA1 is also expressed by sensory neurons and was initially described as a noxious cold sensor with an activation temperature of 17°C [15]. However, many reports have indicated that TRPA1 is not directly gated by cold [16]–[19] or may only be weakly activated after prolonged exposure to cold temperatures [20]–[22]

We reasoned that cold-activated TRP channels in primary afferent neurons might play an important role in the pathogenesis of cold allodynia. An increased expression of these channels or an alteration in their functional properties could lead to the lower thresholds for cold pain and increased sensitivity to cold seen in neuropathic pain states. We therefore examined the expression and function of TRPM8 and TRPA1 in DRG neurons in a mouse model of neuropathic pain. We demonstrate that the TRPM8 agonist menthol evokes nociceptive behavior after nerve injury, and that TRPM8 is a potential transducer molecule for cold-mediated allodynia. However, we find no evidence of increased expression of TRPM8 and TRPA1, or of changes in the cold sensitivity of sensory neurons after injury.

## Results

### Behavioral responses to cold and menthol

**Figure 1:**
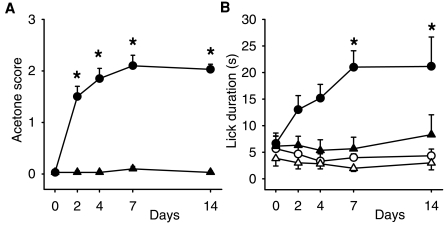
Time course of cold and (-)-menthol sensitivity following sciatic nerve ligation. (a) Cold sensitivity assessed by acetone response score where 0 = no response, 0.5 = licking, 1 = flinching and brushing of the paw, 2 = strong flinching, 3 = strong flinching and licking. Circles, CCI operated animals (n = 6) and triangles, control animals (n = 6). *P<0.05 CCI against control, two-way repeated measures ANOVA followed by Student-Newman-Keuls test. (b), Menthol evoked paw licking duration. Filled circles, CCI operated mice treated with (-)-menthol (250 mM). Open circles, CCI mice with vehicle (90%DMSO, 10% PBS). Filled triangles, control mice (-)-menthol. Open triangles, control mice vehicle. All values are mean±SEM. *P<0.05 CCI (-)-menthol against control (-)-menthol, two-way repeated measures ANOVA followed by Student-Newman-Keuls test.

We used a chronic constriction injury of the sciatic nerve to model neuropathic pain. Cold allodynia is prevalent in this model [23] and using the acetone test we observed robust behavior such as licking, brushing and flinching of the paw indicative of cold allodynia (Fig. 1a).

The ion channel TRPM8 is the best candidate cold transduction molecule that has been identified to date [12]–[14]. We therefore investigated whether TRPM8 activation might be important for cold allodynia. We reasoned that if TRPM8 is a receptor for cold-evoked allodynia after injury, then activation of the ion channel with its agonist menthol should also evoke nociceptive behavior.

We applied 40 µl of (-)-menthol or vehicle to the ipsilateral paw of CCI or control mice and monitored behavioral responses (Fig. 1b). Vehicle application did not evoke nociceptive behavior in CCI or control mice. Application of (-)-menthol in control mice led to a small increase in licking duration compared to vehicle, but this was not statistically significant (P = 0.0956, two-way repeated measure ANOVA). However, application of (-)-menthol in CCI mice led to a significant increase in licking duration (P<0.001 compared to (-)-menthol control mice, two-way repeated measure ANOVA) that became apparent 2 days after injury and peaked 7 days after injury, paralleling the development of cold allodynia in the acetone test. In addition to licking of the treated paw, CCI mice also displayed behaviors such as flinching of the paw and brushing of the affected area that also developed with a similar time course to cold-evoked behavior. This suggests that activation of TRPM8 after nerve injury, either by (-)-menthol or cold, can evoke nociceptive behavior.

### TRPM8 and TRPA1 expression levels after nerve injury

**Figure 2:**
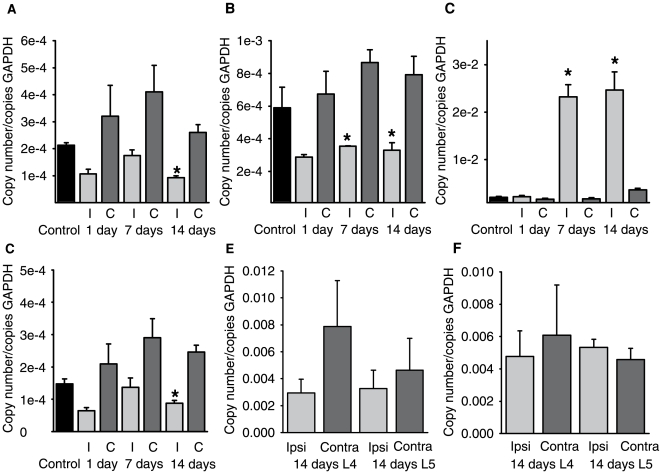
Quantitative reverse-transcription PCR. mRNA levels for (a), TRPM8 (b), TRPA1 (c), galanin, and (d) TREK-1 in the mouse. Levels are expressed relative to GAPDH in control animals and at 2, 7 and 14 days after surgery. I indicates ipsilateral to the injury, C, contralateral. (e) qRT-PCR for TRPM8 and (f) TRPA1 in the rat at 14 days post injury. L4 and L5 indicate respective ganglia. *P<0.05 ipsilateral versus contralateral, paired T-test. All values are mean±SEM (n = 6 animals for each group).

Injury-induced alterations in the number of cold transduction molecules in sensory neurons could underlie the development of cold allodynia. We therefore examined gross expression levels of TRPM8 and TRPA1 mRNA in DRG of injured neurons using qRT-PCR. We detected a relatively low level of mRNA for TRPM8 and TRPA1 in the DRG of control mice (TRPM8, 106400 copies/µg total RNA, TRPA1, 280900 copies/µg RNA) which correlates well with the selective expression of these channels within small subpopulations of neurons. After injury, the expression level of the channels was slightly decreased, reaching statistical significance on day 14 for TRPM8 (Fig. 2a) and on day 7 and 14 for TRPA1 (Fig. 2b). We investigated the relevance of these small reductions by comparing them to the expression of the neuropeptide galanin as a positive control. Galanin has been shown to be strongly up-regulated after nerve injury [24] and indeed in our experiments we measured a considerable increase in galanin mRNA levels at 7 and 14 days post CCI (Fig. 2c). This demonstrates that the CCI model and qRT-PCR technique used here are feasible methods for measuring injury induced alterations in mRNA levels.

Because TRPM8 and TRPA1 displayed a reduced expression after injury we reasoned that another cold-sensitive ion channel might be upregulated instead. We thus performed qRT-PCR experiments on the background K^+^ channel TREK-1 which has been demonstrated to be thermosensitive [25]. However, similar to TRPM8 and TRPA1, this mRNA was also expressed at a lower level after injury (Fig. 2d).

It is also possible that changes in cold channel mRNA expression levels are species dependent. We therefore examined TRPM8 and TRPA1 expression in the rat after CCI injury. We also investigated changes in individual L4 and L5 ganglia to ensure that pooling of L3-L5 ganglia was not masking a potential increase in expression. However in agreement with our results from mice, TRPM8 and TRPA1 did not show upregulation after nerve injury (Fig. 2e and f).

### Distribution of TRPM8 and TRPA1 mRNA after nerve injury

We examined the expression of TRPM8 and TRPA1 mRNA in more detail using in situ hybridization of mouse DRG sections. This technique allowed us to identify the neuronal subtype expressing each channel.

To this end we employed a double-labeling technique utilizing antibodies against calcitonin gene related peptide (CGRP) and neurofilament 200 kDa (NF200) to identify peptidergic nociceptors and myelinated neurons, respectively, and fluorescently labeled IB4 to detect non-peptidergic nociceptors [26]–[28]

**Figure 3:**
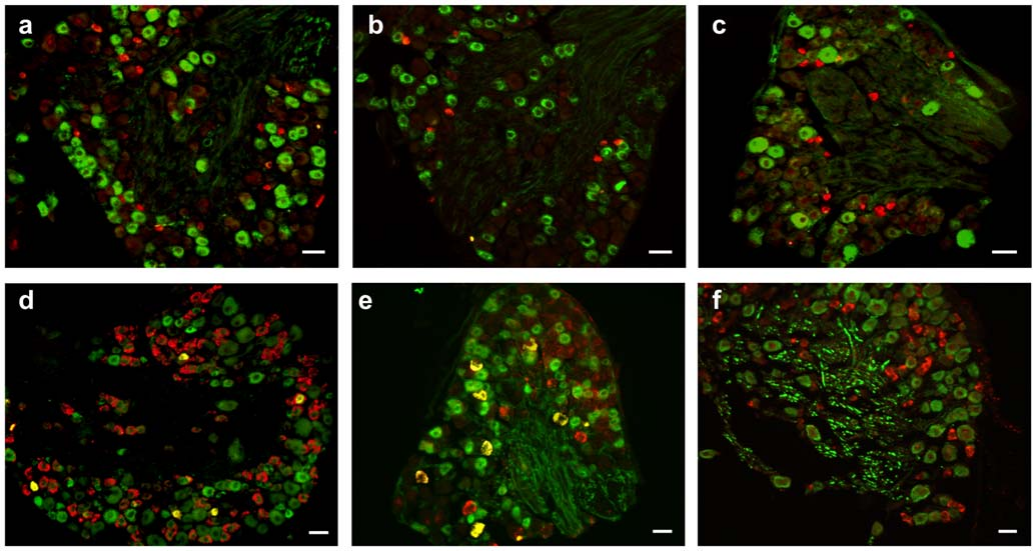
Distribution of TRPM8 and TRPA1 in identified neuronal subpopulations in DRG. Combined in situ hybridization of TRPM8 (red) with (a), immunohistochemistry (green) for CGRP, (b), IB4 and (c), NF200. TRPA1 mRNA expression (red) with (d), CGRP, (e), IB4 and (f), NF200 (green). Scale bar 40 µm.

We detected TRPM8 mRNA in approximately 7% of neurons within the DRG. This was reduced to ∼5%, 7 and 14 days post injury (Table 1). TRPM8 was co-expressed in very few CGRP-positive peptidergic neurons (Fig. 3a) and essentially no IB4 positive non-peptidergic neurons (Fig. 3b) or NF200 containing myelinated neurons (Fig. 3c). Co-expression of TRPM8 with any of the markers did not change after injury (Table 1).

**Table 1:** Distribution of TRPM8 mRNA in the DRG after nerve injury.

	TRPM8 mRNA	TRPM8 + CGRP	TRPM8 + IB4	TRPM8 + NF200
Control	6.6±0.4 (796/12276)	0.2±0.1 (8/5283)	0 (2/4356)	0 (0/2637)
7 day Ipsi	4.5±0.3* (638/14523)	0.1±0.1 (6/3047)	0 (1/5998)	0 (1/5478)
14 day Ipsi	4.6±0.2* (725/15947)	0.2±0.1 (12/7621)	0 (0/4790)	0 (1/3536)

Table showing the percentage (±SEM, n = 6 mice per group) of neurons in the DRG expressing TRPM8 alone and co-localized with CGRP, IB4 or NF200. Numbers in brackets indicate the total number of neurons counted.

*P<0.05 compared to control, T-test.

TRPA1 mRNA expression was also significantly reduced following nerve injury (Table 2). The majority of TRPA1 positive cells were co-labeled with IB4, with additional expression in some CGRP-expressing neurons and almost no expression in NF200-labeled cells (Fig. 3d–f, Table 2). Intriguingly the reduction in TRPA1 expression was seen exclusively in the IB4-positive neurons, with no change in the number of TRPA1 plus CGRP or TRPA1 plus NF200 positive cells (Table 2).

**Table 2:** Distribution of TRPA1 mRNA in the DRG after nerve injury.

	TRPA1 mRNA	TRPA1 + CGRP	TRPA1 + IB4	TRPA1 + NF200
Control	28±1.2 (2678/9958)	2.5±0.4 (66/2688)	25.3±1.9 (766/2805)	0.6±0.2 (21/4465)
7d Ipsi	19.6±2.2* (1642/8514)	2.1±0.3 (57/2636)	19±1.7* (484/2089)	0.7±0.1 (29/3789)
14d Ipsi	20.7±1.7* (1763/8834)	3.1±0.3 (154/4914)	17.4±2.2* (422/1729)	0.7±0.2 (17/2191)

Table showing the percentage (mean±SEM, n = 6 mice per group) of neurons in the DRG expressing TRPA1 alone and co-localized with CGRP, IB4 or NF200. Numbers in brackets indicate the total number of neurons counted.

*P<0.05 compared to control, T-test.

In order to study the regulation of TRPA1 mRNA in another model, we also examined its expression during inflammatory pain. We injected Complete Freund's Adjuvant (CFA) into the hind paw to induce inflammation and assessed the expression of TRPA1 mRNA 48 hours later. In contrast to the reduction in expression seen following nerve injury, we found no difference in the number of cells expressing TRPA1 after CFA injection (Table 3).

**Table 3:** Distribution of TRPA1 mRNA in the DRG after inflammation.

	TRPA1 mRNA	TRPA1 + CGRP
Control	31.3±1.3 (2738/8558)	3.18±0.4 (238/8558)
48 hours CFA	32.04±0.9(4071/12601)	3.69±0.31 (433/12601)

Table showing the percentage of neurons in the DRG expressing TRPA1 alone and co-localized with CGRP in control and CFA injected mice (±SEM, n = 3 mice per group). Numbers in brackets indicate the total number of neurons counted.

Our in situ hybridization data is in broad agreement with our qRT-PCR data and shows that TRPM8 and TRPA1 mRNA are down-regulated in the DRG following nerve injury. Furthermore, there is no evidence of de novo synthesis of channel mRNA amongst the different subpopulations of sensory neurons, suggesting that a “phenotypic switch” in cold-sensing neurons is not a mechanism for cold allodynia.

### Functional properties of cold-sensitive sensory neurons after nerve injury

We used Ca^2+^ microfluorimetry from acutely isolated mouse DRG neurons to measure functional properties of cold-sensitive neurons after nerve injury. TRP channel expression remains constant for a period of hours after dissociation [29], therefore all experiments were performed within 3 hours of plating cells.

**Figure 4:**
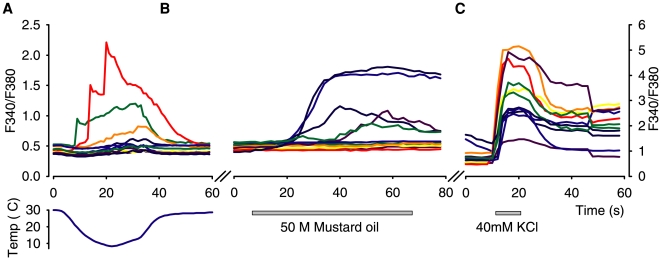
Representative recordings of Ca^2+^ transients in DRG neurons from control mice. Responses to (a), cooling, (b), mustard oil and (c), KCl (note different Y-axis scale for KCl).

Our aim here was to determine the number of cold responsive cells in the DRG and to identify whether they contained TRPM8 or TRPA1 as potential transduction molecules. Figure 4 shows a typical experiment in which sensory neurons were tested for responses to cold, mustard oil (a TRPA1 agonist) and KCl. In this example, 3 of 12 neurons responded robustly to cold, 5 of 12 responded to mustard oil (and therefore presumably expressed TRPA1), and 1 of 12 cells responded to both stimuli. Similar experiments were performed using (-)-menthol to identify TRPM8 positive neurons.

**Figure 5:**
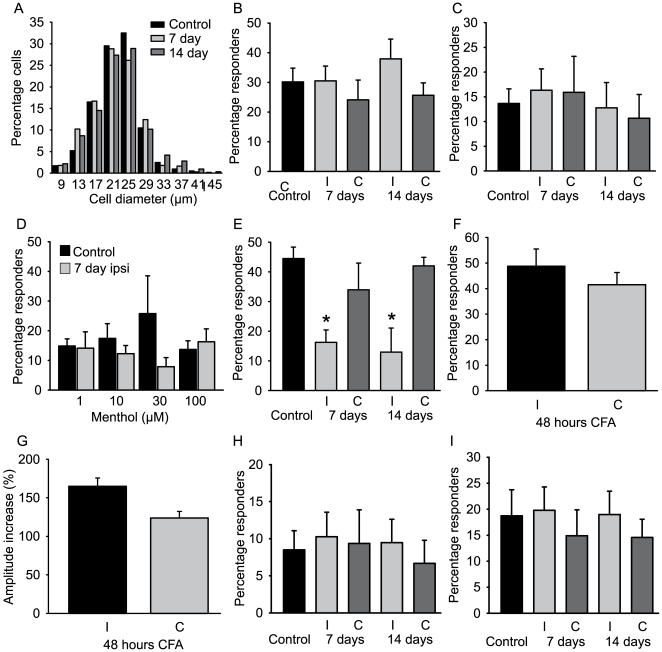
Proportions of cold, (-)-menthol and mustard oil responsive DRG neurons in control animals, at 7 and 14 days post-CCI and at 48 hours post CFA. (a), Cell-size histogram in control mice and in mice at 7 and 14 days post CCI injury. (b), Percentage of cold-responsive neurons in the DRG (n = 6–15 mice). (c), Percentage of neurons responsive to 100 µM menthol (n = 6–15 mice). (d), Concentration-response profile for menthol. (e), Percentage of mustard oil-responsive neurons (n = 3 mice in each group). (f), Percentage of neurons responding to mustard oil in DRG ipsilateral and contralateral to CFA injection (n = 3 mice). (g), Maximum amplitude of Ca^2+^ transients in mustard oil sensitive neurons following CFA injection. (h), Percentage of neurons in the DRG responsive to both (-)-menthol and cold (n = 6–11). (i), Percentage of neurons in the DRG insensitive to (-)-menthol but responsive to cold (n = 6–11). I indicates ipsilateral to the injury, C contralateral. *P<0.05 ipsilateral versus contralateral, paired T-test. All values are mean±SEM.

We analyzed a total of 3655 cells from control, 7 day and 14 day post-injury mice. Cells sizes had a similar distribution in each group indicating that we were not sampling from different populations (Fig. 5a). In control animals we found that 30±5% of all DRG neurons responded to cooling, 14±2% responded to 100 µM (-)-menthol and 45±2% of cells responded to mustard oil (Fig. 5b–e). After nerve injury, we observed no change in the proportions of cells responding to cold or to a range of concentrations of (-)-menthol (Fig. 5b, c, d). However, in agreement with our expression analysis, the number of cells responding to mustard oil was strongly reduced at days 7 and 14 post CCI (Fig. 5e) (although the amplitude of individual responses was not changed). Additionally, we investigated mustard oil responses in the CFA model of inflammation and at 48 hours post injection there was no change in the number of cells responding to mustard oil (Fig. 5f). However, we did observe significantly larger mustard oil evoked responses in CFA-treated inflamed mice compared to control mice (Fig. 5g).

Our Ca^2+^ imaging analysis allowed us to define different subpopulations of cold-responsive neurons based upon their (-)-menthol and mustard oil sensitivity. We observed a strong association between (-)-menthol sensitivity and cold activation such that 72±8 percent of all (-)-menthol responding neurons were also responsive to cold (∼9% of all neurons in the DRG responded to both (-)-menthol and cold and this value did not change after injury (Fig. 5h)). In contrast, mustard oil and cold sensitivity showed little correlation. Only 18±2% of mustard oil sensitive neurons responded to cold (8±1% of all DRG neurons) and this value was further decreased 7 days following injury (2±1% of all neurons). The lack of overlap between mustard oil and cold sensitivity argues against a role for TRPA1 in cold sensation both in control animals and following nerve injury. We therefore continued our analysis by classifying cold sensitive neurons as either menthol-sensitive or menthol-insensitive (Fig. 5i).

Sensory neurons can be classified by their cell size with smaller neurons (<25 µm in the mouse) being predominantly nociceptive. We measured cell soma diameters in cold-sensitive cells and observed an average value of 18.8±0.4 µm in the menthol-sensitive population and 19.5±1.1 µm in the menthol-insensitive population. Mustard oil responsive neurons were significantly larger with a diameter of 23±1.3 µm (p<0.05 Mann-Whitney-U-test) demonstrating the lack of functional overlap with cold sensing neurons. None of these values changed after nerve injury supporting our expression analysis and confirming that a novel population of cold sensory neurons does not emerge after CCI.

We also measured functional properties of cold-responsive neurons such as their activation temperature thresholds and the maximum amplitude of response to cold, menthol and mustard oil. Table 4 shows that temperature thresholds were similar in menthol-sensitive and menthol-insensitive neurons and that this value did not change after CCI. The size of the response was significantly larger in menthol-sensitive and mustard oil responsive neurons compared to menthol-insensitive cold neurons (P = 0.02 and P = 0.01 respectively, two-way ANOVA), although again, this was not altered post injury.

**Table 4:** Temperature threshold and amplitude of Ca^2+^ transients in DRG neurons after nerve injury.

	Temperature threshold		Maximum response		
	MS	MI	Cold MS	Cold MI	Mustard Oil
Control	20.3±0.9°C	18±2°C	110.9±17.2%	58.9±15.1%	108.1±16.7%
7 day Ipsi	19.4±2°C	16±1.3°C	116.3±26.1%	51.4±7.13%	92.1±7%
14 day Ipsi	21.5±2°C	16.3±1.1°C	66.6±21.6%	43.9±5.8%	86.6±28.8%

Table showing the temperature threshold and maximum amplitude of Ca^2+^ responses (percent increase from baseline) in menthol sensitive (MS) and menthol insensitive (MI) cold neurons and in mustard oil responsive neurons (mean±SEM).

## Discussion

In the present study we explored the role of the ion channels TRPM8 and TRPA1 in the development of cold allodynia following nerve injury. We show that menthol evokes nociceptive behavior after injury that parallels the emergence of cold-evoked hypersensitivity. We examined the expression of TRPM8 and TRPA1 in the DRG, and analyzed the receptive properties of cold sensitive sensory neurons post-injury. Unexpectedly, we observed a reduction in the expression of the TRP channel mRNA and no difference in the functional properties of cold sensitive DRG cells. Our results suggest that cold allodynia does not arise directly from changes in TRP channel expression.

### Menthol evokes nociceptive behavior post-injury

The psychophysical effects of menthol have been well documented in humans. Menthol is known for its ability to evoke a cooling sensation when applied to the skin [30]–[32], and high concentrations (30–40% w/v) produce burning pain and cold hyperalgesia [33]–[35]. There are fewer studies addressing the in vivo effects of menthol in animal models, presumably because of the difficulty in designing behavioral assays to assess innocuous stimuli, and because higher nociceptive concentrations of menthol induce ataxia when injected in mice [14].

In our experiments, we applied 250 mM (3.9% w/v) (-)-menthol to a localized area of the hind paw. We observed no ataxia and indeed control mice behaved in much the same way as vehicle treated animals. After nerve injury however, the same concentration of (-)-menthol evoked strong nociceptive responses that developed with a time course similar to acetone-induced cold behavior. This indicates that activation of TRPM8 in CCI mice triggers nociceptive behavior and that TRPM8 might be a key component of cold hypersensitivity. In agreement, Colburn et al. (2007) reported that TRPM8 knockout mice display reduced acetone responses after nerve injury, suggesting that in neuropathic pain states TRPM8 acts as the predominant sensor for cool-evoked pain.

Menthol is also known to have analgesic actions in a number of painful conditions. A recent report demonstrated that activation of TRPM8 by (-)-menthol and other agonists led to marked analgesia in the CCI model of neuropathic pain in rats [36]. One major difference between that study and ours was that considerably lower concentrations of (-)-menthol were used (4 mM compared to 250 mM used here). It is therefore likely that low concentrations of (-)-menthol (4 mM) evoke anti-nociception, while higher concentrations (250 mM) are pro-nociceptive after injury. Intriguingly, in healthy human volunteers, concentrations similar to the one used here (320 mM–630 mM) induce a cooling sensation [30], [32], whereas concentrations of 1.9 M–2.6 M are required to evoke pain [33]–[35]. An important question is whether the concentration of menthol required to evoke pain is also lowered in neuropathic pain patients.

### Nerve-injury induced changes in the expression of TRPM8 and TRPA1

Several studies have examined the expression of TRPM8 and TRPA1 mRNA in rat models of neuropathic pain. mRNA transcripts were reported to be weakly up-regulated in DRG when measured using a ribonuclease protection assay [37], and TRPA1 mRNA was found to be down-regulated in injured neurons, but up-regulated in uninjured neurons in the L5 spinal nerve ligation (SNL) model [38]. However, using the same model, another study reported down-regulation of both TRPA1 and TRPM8 in injured neurons and no change in expression in the uninjured ganglion [39]. At the protein level, the number of cells expressing TRPM8-immunoreactivity is increased in the CCI model [36], [40]. Interestingly, we observed that TRPA1 was predominantly expressed in IB4 positive neurons and that it was in this population of neurons that TRPA1 expression was lost after injury. Previous studies have demonstrated that IB4 binding is strongly reduced in injured neurons following SNL in the rat [41] suggesting that loss of this population contributes to the reduction in TRPA1 expression.

One common feature of these studies and of our results is that injury induced changes in the expression of TRPA1 or TRPM8 were relatively minor, especially when compared to molecules such as galanin. Moreover, Obata et al. (2005) reported that TRPA1 up-regulation was only detected in uninjured neurons in the SNL model. It was argued that this selective increase in TRPA1 expression in the uninjured L4 DRG underlied the emergence of cold hypersensitivity. However, we and others [39] detected no up-regulation of TRPA1 in either injured or uninjured neurons after nerve injury, but were still able to measure robust cold allodynia.

We also considered the possibility that species differences between rats and mice might account for this discrepancy. We repeated our qRT-PCR experiments in rats and took the additional step of measuring from individual L4 and L5 ganglia to increase sensitivity. However, in agreement with our results in mice, we were unable to detect an upregulation in TRP channel expression in either ganglia following injury. This suggests that cold hypersensitivity in neuropathic pain is not always dependent upon increased expression of TRPA1 and TRPM8 in DRG.

### Cold sensitivity of sensory neurons after nerve injury

We identified TRPM8 positive neurons by applying (-)-menthol to dissociated DRG cells. Interestingly, the number of cells responding to 100 µM menthol in culture was greater than expected from our expression analysis and about a quarter of all menthol-sensitive neurons did not respond to cold. One explanation for this is that menthol is not selective for TRPM8. Indeed, menthol has been demonstrated to evoke Ca^2+^ release from intracellular stores via a TRPM8-independent pathway [42], and to activate TRPA1 at lower concentrations [43], [44]. We observed a similar trend in experiments where we used lower concentrations of menthol (Fig. 5d). For example at 30 µM menthol, a reduction in the number of responders was evident in neuropathic animals, paralleling our TRPA1 results. Thus a TRPM8 independent mechanism could account for some of the menthol responsive cells observed here.

TRPA1 containing neurons were identified by their response to mustard oil and in these cells we observed no correlation between cold and mustard oil sensitivity. We applied cold stimuli as low as 8°C (well below the proposed temperature threshold for TRPA1 [15], [21]) but were unable to activate the majority of mustard oil sensitive neurons (over 80%). It is possible that the duration of our cold stimulus (30 seconds) was not sufficient to activate TRPA1 since it has been reported that prolonged cooling is required to evoke a response [22]. We recently reported that TRPA1 is activated by intracellular Ca^2+^ and that cold sensitivity occurs indirectly through an increased Ca^2+^ concentration in HEK293 cells [19]. However, cold does not evoke a general increase in Ca^2+^ concentration in sensory neurons (as occurs in HEK293 cells), thus even an indirect role for TRPA1 in acute cold sensation seems unlikely. Consequently, we did not examine behavioral responses to mustard oil in CCI mice and we continued our functional analysis by designating cold responsive neurons as either menthol-sensitive, or menthol-insensitive as has been reported in previous studies [45]-[47].

Intriguingly, we found that the number of mustard oil responsive cells was strongly reduced after CCI but not changed following CFA injection. It is difficult to gauge the functional significance of TRPA1 down-regulation and its contribution to the pathophysiology of neuropathic pain. However, voltage-gated Ca^2+^ currents are also diminished after nerve injury [48] and this has been proposed to lead to increased excitability in injured sensory neurons via reduced activation of Ca^2+^ activated K^+^ currents. Since TRPA1 is gated by, and is permeable to Ca^2+^ [19], it is possible that reduced expression of the channel might also contribute to diminished Ca^2+^ currents and enhanced excitability after injury. We also measured the amplitude of responses to mustard oil in neuropathic and inflammatory pain models. In CCI animals there was no alteration, however CFA injection resulted in an increased amplitude. Again this could reflect a change in basal intracellular Ca^2+^ concentration in TRPA1 positive neurons during inflammatory pain. An increased level of Ca^2+^ via activation of Ca^2+^ signaling pathways by inflammatory mediators would serve to increase the sensitivity of TRPA1 to its agonists [19].

A surprising aspect of our results is that we observed no differences in any parameters of cold sensitivity post-injury. This is in contrast to some studies examining cold sensitivity in the SNL and CCI models or neuropathic pain [40], [49], [50]. Using the SNL model, it was reported that while cold responses remained constant in injured neurons, a proportion of uninjured neurons of the L4 DRG developed sensitivity to cold after injury. Similarly, in the CCI model a small population of capsaicin-sensitive neurons developed cold sensitivity after injury. These novel populations of cold sensing neurons could potentially contribute to cold allodynia, however our data suggests that this is unlikely to be a universal mechanism for cold hypersensitivity. In support of this, using an experimental neuroma model in mice, Roza et al. [51] demonstrated that cold sensitivity of axotomized fibers does not change from pre-injury levels.

Our data indicate that an alteration in the number of cold sensitive DRG neurons is not a mechanism for cold allodynia in the CCI model of neuropathic pain. However, it does not exclude the possibility that changes in cold sensitivity might occur in the peripheral terminals of sensory neurons. For example, it is possible that CCI alters the transport of cold sensitive channels to the periphery leaving a deficit of mRNA at the level of the DRG. Similarly, alterations in cellular context could shape the response of cold sensitive neurons at their peripheral terminals. Recently Madrid et al. [52] demonstrated a correlation between expression level of I_KD_ (a current dependent upon Shaker-like Kv1 channels) and threshold of activation by cold. Blocking I_KD_ shifted the activation threshold of noxious cold activated neurons to higher temperatures and it was argued that a similar mechanism could account for cold allodynia.

In the absence of a peripheral mechanism for cold allodynia, a modification of central processing of cold stimuli after nerve injury could underlie the emergence of cold hypersensitivity. In a recent fMRI study of healthy human volunteers, it was reported that cold allodynia induced by 40% menthol, recruited the bilateral dorsolateral prefrontal cortex and the midbrain to cold pain processing, regions not normally associated with cold sensation [53].

Changes at the spinal cord level could also trigger cold allodynia and several spinal mechanisms such as structural reorganization, central sensitization of dorsal horn neurons, and disinhibition of nociceptive circuitry have been postulated to contribute to neuropathic pain states [1]. Interestingly, a number of human studies have shown that cold pain sensation is under tonic inhibitory control. Thus blockade of myelinated fiber conduction during cooling has been demonstrated to abolish innocuous cold sensation and induce burning and stinging pain rather than coolness [54]. Similarly, fibers transmitting innocuous cold might also exert inhibition, a hypothesis that is supported by the observation that some neuropathic pain patients are unable to perceive cold but report burning pain on cold exposure [55]. Intriguingly, the central terminals of TRPM8 expressing neurons have been demonstrated to overlap with glutamic acid decarboxylase 65 (GAD65) in lamina I of the spinal cord [56]. GAD65 synthesizes the inhibitory enzyme GABA, suggesting that TRPM8 positive cold sensory neurons could form contacts with inhibitory dorsal horn neurons. From our results we are unable to identify the central mechanism of cold hypersensitivity, however it is interesting that we observed decreases in TRPM8 expression. If this were to occur selectively in cool sensing neurons involved in the suppression of cold pain, it could lead to an unmasking of cold nociceptive pathways. A shift in the balance of inhibitory to excitatory input would allow TRPM8 activation, either by menthol or cold, to evoke pain.

## Materials and Methods

### Surgical procedures

All experiments were conducted on C57/B6 mice or Wistar rats with the approval of the state animal care and use committee (Landesamt für Arbeitsschutz, Gesundheit und Technische Sicherheit Berlin). A chronic constriction injury (CCI) of the sciatic nerve was used to model neuropathic pain. Briefly, the right sciatic nerve was exposed at mid-thigh level under isoflurane anesthesia. 3 loose silk ligatures were tied around the nerve and the incision was closed. For sham controls, the sciatic nerve was exposed but not ligated.

Intraplantar injection of Complete Freunds Adjuvant (CFA) was used to model inflammatory pain. 20 µl CFA (50 µg of desiccated M. Butyricum diluted into 20 µl Incomplete Freunds Adjuvant from Difco Laboratories Detroit, USA) was injected into the right hind paw under brief isoflurane (Rhodia Organic Fine Ltd., Bristol, UK) anesthesia. The inflammation was confined to the right paw throughout the observation period. Animals were killed after 48 hours and tissue was prepared as described below.

### Behavioral experiments

For all behavioral experiments, mice were habituated to the test procedure for 7 days before surgery. Responses were taken 1 day prior to surgery and at 2, 4, 7 and 14 days postoperative.

The acetone test was used to assess cold allodynia in mice. In this test, evaporative cooling of locally applied acetone is used to evoke nociceptive behavior in CCI mice [57], [58]. 40 µl of acetone was applied to the dorsal hind paw ipsilateral to the injury and the behavior was assigned an arbitrary score. A score of 0 indicated no response, 0.5, a licking response, 1, flinching and brushing of the paw, 2, strong flinching, and 3, strong flinching and licking. Behavior was observed during the first 30 seconds after acetone application and measurements were repeated 2 times with a 1 minute interval to obtain a mean value. Dorsal root ganglia from animals displaying cold allodynia were used for qRT-PCR, in situ hybridization and calcium imaging assays.

To assess the effects of menthol on nociceptive behavior in CCI mice, 40 µl of 250 mM (-)-menthol (Sigma, Germany) dissolved in 90%DMSO and 10%PBS or vehicle alone was applied to the dorsal surface of the ipsilateral hind paw. Mice were observed for 5 minutes and the time spent licking the hind paw was measured. Experiments were repeated in control mice.

### Quantitative reverse transcription-PCR

Lumbar L3-L6 dorsal root ganglia (DRG) were dissected from CCI and control mice, pooled and total RNA extracted using the RNeasy mini kit (Qiagen, Germany). Samples were quantified using a spectrophotometer (Gene Quant II, Pharmacia Biotech, UK) and reverse transcribed with AMV-reverse transcriptase (Roche, Germany). Quantitative PCR was performed using the Lightcycler system (Roche, Germany) utilizing SYBR green to detect amplification. PCR primers were designed to amplify 400 bp regions from TRPM8, TRPA1, TREK-1, galanin and the housekeeping gene GAPDH as a reference. These primers were tested at a range of annealing temperatures and were found to amplify 1 PCR product as determined by melting curve analysis and agarose gel electrophoresis. A standard concentration curve for each cDNA was constructed from serial dilutions of linearized plasmid DNA. Experiments were performed in triplicate and data was normalized to GAPDH levels.

In further experiments, individual L4 and L5 ganglia were dissected from control and CCI rats and processed as above for qRT-PCR of TRPM8, TRPA1 and GAPDH mRNA.

### In situ hybridization and immunohistochemistry

Mouse tissue was fixed by transcardial perfusion of 4% paraformaldehyde (PFA) in PBS. Lumbar L3-L6 DRG were dissected, post-fixed for a further 2 hours in PFA and cryoprotected overnight in 30% sucrose. Non-radioactive in situ hybridization was performed using digoxygenin (DIG) (Roche, Germany) labeled RNA probes on 10 µm frozen sections. Antisense probes corresponded to nucleotides 562–1465, 262–808, 673–1087 and full length for TRPA1 and 963–1533, 242–870 and full length for TRPM8. Equivelant sense probes displayed no signal.

Sections were treated with 1 µg/ml proteinase K (Sigma, Germany) for 5 minutes, acetylated for 10 minutes with 0.25% (v/v) acetic anhydride in 0.1 M triethanolamine, prehybridized for 4 hours at 56°C and hybridized with RNA probes overnight at 56°C. Following post hybridization washes and blocking, sections were incubated for 30 minutes in 1∶100 anti-DIG antibody conjugated with horseradish peroxidase (Roche, Germany) and signal was visualized using tyramide signal amplification (PerkinElmer, Germany). Immunohistochemistry and isolectin B4 (IB4) staining followed in situ hybridization. FITC-labeled IB4 (Sigma, Germany) was used at a concentration of 10 µg/ml, anti-CGRP (polyclonal, Sigma, Germany) at a 1∶2000 dilution, and anti-NF200 (clone N52, Sigma, Germany) at 1∶4000 dilution. All cell counts and quantification were conducted by an observer blinded to the experimental condition.

### Calcium imaging

Mouse lumbar L3-L6 DRG were dissected from CCI and control mice and incubated with 1 mg/ml collagenase IV (Sigma, Germany) and 0.05% trypsin (Biochrom, Berlin, Germany) for 30 minutes each at 37°C. The DRG were suspended in DMEM/Hams-F12 medium (Invitrogen, Germany) containing 10% heat-inactivated horse serum (Biochrom, Berlin, Germany), 1 mM glutamine (Invitrogen, Germany) 0.8% glucose (Sigma, Germany), 100U penicillin, and 100 µg/ml streptomycin (Biochrom, Berlin, Germany). DRG were dissociated using 18G, 22G, 25G needles, and debris was removed with a 40 µm cell strainer (BD Biosciences Europe, Belgium). Cells were plated in a droplet of medium on poly-L-lysine (100 µg/ml, Sigma, Germany) coated coverslips and left to adhere for 30 minutes before the coverslip was flooded. Experiments were conducted 1–3 hours after plating of cells.

Ratiometric calcium imaging was performed with FURA-2/AM dye (Invitrogen, Germany) and analyzed using Tillvision software (Till Photonics, Germany). Cells were loaded with 3 µM Fura-2/AM and placed in a recording chamber containing calcium imaging buffer (CIB: 140 mM NaCl, 4 mM KCl, 2 mM CaCl_2_, 1 mM MgCl_2_, 5 mM Glucose, 10 mM HEPES, pH 7.4). Pairs of images were collected every 2 seconds at alternating exposures of 340 nM and 380 nM (exposure time 70 ms) using a Polychrome V monochromator and CCD Imago camera (Till Photonics, Germany). Following subtraction of background fluorescence the ratio of fluorescence at 340 nm and 380 nm was calculated.

Coverslips were superfused with CIB buffer at approximately 2 ml/min. Drugs were applied via a gravity driven perfusion system that allowed rapid exchange of solutions. Cold stimuli were applied using a peltier device (ESF electronic, Germany) and temperature changes were monitored with a thermocouple placed within the flow of buffer and close to the cells.

Following selection of a suitable field of view, coverslips were maintained at 31°C for 5 minutes. A cold stimulus was then applied which cooled the cells from 31°C until an endpoint of 8°C had been reached. Cooling occurred at approximately 1°C per second and was reproducible such that in more than 95% of experiments the cold endpoint was reached within 30 seconds. After a recovery period of 5 minutes at 31°C, either (-)-menthol (100 µM unless indicated), or in separate experiments, mustard oil (50 µM based on dose response date (not shown)) was applied for 1 minute. Drugs were washed out for 3–5 minutes before 40 mM KCl was applied for 10 seconds to determine the total number of living cells.

A response was designated as a 20% increase in fluorescence ratio from baseline. The number of responders to cold, (-)-menthol or mustard oil was expressed as a percentage of KCl responsive cells. The maximum amplitude of the response, the temperature threshold of cold responses and the cell diameter were also determined. Cold threshold temperatures were calculated as the temperature at which fluorescence ratio increased by 0.5% upon cooling. These relatively stringent selection criteria for cold responsive cells plus the fact that we started our recordings at 31°C (rather than 35–37°C used by some researchers) may account for the low temperature thresholds we observed.

### Data analysis

Statistical analysis was performed using Sigmastat software (Systat, San Jose, CA). Significance was tested using a two-way repeated measures ANOVA in behavioral experiments, and a Student's T-test or Mann-Whitney-U-test in expression and calcium imaging experiments.
